# Risk factors for predicting increased surgical drain output in patients after anterior cervical corpectomy and fusion

**DOI:** 10.1186/s13018-017-0698-5

**Published:** 2017-12-28

**Authors:** Jinqian Liang, Jianhua Hu, Chong Chen, Hao Yin, Fangliang Dong

**Affiliations:** 1 Department of Orthorpaedic Surgery, Peking Union Medical College Hospital, No.1 Shuaifuyuan, Dongcheng district, Beijing, 100730 People’s Republic of China; 2 Department of Spine Union, Hunan Provincial People’s Hospital, No.61 Jiefangxi Road, Changsha, Hunan 410005 People’s Republic of China; 3 Department of Spine Union, Puyang Anyang Area Hospital, No.260 Dengta Road, Anyang, Henan 455000 People’s Republic of China

**Keywords:** Anterior cervical corpectomy and fusion, Retrospective study, Risk factors, Surgical drain output

## Abstract

**Background:**

Although measures to reduce and treat the postoperative surgical drain output are discussed, along with the increased interest in causative factors related to the prevention and treatment reported by many studies, these are still controversial.

**Methods:**

A retrospective study was conducted on a consecutive series of 217 patients who had underwent ACCF between January 2016 and March 2017. Patients were categorized based on normal or increased total drain output. These two groups were compared for demographic distribution and clinical data to investigate the predictive factors of increased drain output by multivariate analysis.

**Results:**

The overall incidence rate of increased drain output after ACCF was 16.6%. There are no significant differences in sex, BMI, history of taking aspirin, and ASA classification between the two groups (*P* > 0.05). Of the patients with increased drain output, a significantly higher proportion of patients have OPLL in the surgical level, 18 (50.0%) versus 33 (18.2%) (*P* = 0.000). The mean age was 60.67 ± 8.18 years versus 54.41 ± 10.05 years (*P* = 0.001). Number of discs involved was 2.42 ± 0.50 versus 2.02 ± 0.65 (*P* = 0.001). Operation time was 112.22 ± 16.49 min versus 105.21 ± 17.89 min (*P* = 0.031). Intraoperative blood loss was 109.86 ± 62.02 mL versus 87.83 ± 56.40 mL (*P* = 0.036). Logistic regression analysis showed that age (OR, 1.075; *p* = 0.003), history of smoking (OR, 2.792; *p* = 0.021), OPLL in surgical level (OR, 2.107; *p* = 0.001), and number of discs involved (OR, 2.764; *p* = 0.003) maintained its significance in predicting likelihood of increased surgical drain output.

**Conclusions:**

The occurrence of increased drain output after ACCF is most likely multifactorial and is related to age, history of smoking, OPLL in surgical level, and number of discs involved.

## Background

Anterior cervical corpectomy and fusion (ACCF) has become a mainstay of treatment for a wide array of cervical pathologies ranging from cervical spondylotic myelopathy (CSM) to deformity correction. While offering the benefit of maximal decompression and minimizing graft interfaces, [1] the procedure is not without drawbacks. In addition to the risks associated with the surgery itself, the blood loss associated with cervical surgery is greater than that with anterior cervical discectomy and fusion (ACDF).

Conventional closed-suction drainage is widely used in spine surgery. In theory, it prevents the formation of hematomas in the operative field, decreases the tension of closed incisions, contributes in avoiding delayed wound healing, and reduces the risk of infection [2, 3]. However, the current literatures in hip and knee arthroplasty research have not shown any additional advantages in such areas as infection, blood loss, changes in hemoglobin and hematocrit, and postoperative function [4–7]. Drainage was also considered unnecessary in the study of orthopedic trauma surgery [8, 9]. Furthermore, some studies have found that active closed-suction drainage is associated with an increase of postoperative wound infection and blood transfusions in spinal surgery [10–12].

Considering the unique and potentially life-threatening complications of neck hematoma (such as airway obstruction), many spine surgeons are reluctant to abandon the use of postoperative drainage in ACCF [13, 14]. Measures to reduce and treat the postoperative surgical drain output are discussed, along with the increased interest in causative factors related to the prevention and treatment reported by many studies. Nevertheless, these are still controversial. Therefore, the identification and quantification of risk factors for increased surgical drain output in patients after ACCF are of paramount importance to the patient and the clinician. In addition to its obvious importance for patient safety, risk factor information becomes critical as health care policy makers implement and enforce “quality” metrics.

This retrospective cohort study was undertaken to investigate (1) the overall incidence of increased surgical drain output in a large population of patients with a background degenerative cervical condition treated with ACCF and (2) the predictive factors for the development of increased surgical drain output in patients after ACCF.

## Methods

We examined data from a consecutive series of 217 patients who had underwent ACCF by one senior surgeon for degenerative cervical disease between January 2016 and March 2017, at one academic hospital—a university-based medical center. This research was approved by the ethics committee of Peking Union Medical College Hospital. Those patients underwent surgery for non-degenerative disease (trauma, infection, tumor, deformity, and inflammation), total disc replacement, concomitant posterior cervical arthrodesis, thoracic or lumbar spine surgery, or other unrelated procedures were excluded from analysis. Those patients who had increased drain output were defined as drain output more than 50 ml. The cohort identified was divided into patients with drain output more than 50 ml and those who did not (control). Drain output was measured and recorded twice a day in 12-h shifts (at 6 a.m. and 6 p.m.). The drain was routinely removed when the drain output per 12-h shift was < 5 mL.

Demographic data included age, gender, smoking history, body mass index (BMI), American Society of Anesthesiologists (ASA) classification, preoperative hematocrit, history of smoking, and history of major medical comorbidities (diabetes mellitus, hypertension, heart disease, or history of taking aspirin). Surgical data collected included number of discs involved and type of bone graft (autograft vs. allograft). In addition, computed tomography (CT) was used to investigate whether there had been an ossification of posterior longitudinal ligament (OPLL) in the surgical level.

The estimated blood loss was based on the use of the blood loss in the suction canister (accounting for irrigation used on the surgical field) plus the blood loss estimated from the difference in weights of dry and blood-soaked sponges. Routine preoperative and postoperative hematocrit were taken from patients’ lab values.

Factors associated with increased drain output were identified using univariate analysis. The data analysis was performed using SPSS version 19.0 (Chicago, IL, USA).Continuous data were compared between the two groups using the student *t* test, whereas discontinuous data were analyzed using the chi-squared test. Fisher’s exact test was used for small data subsets (*n* < 5). All significance tests were two-tailed, with *p* < 0.05 representing statistical significance. In addition, a multivariate logistic regression analysis was performed to identify which factors helped predict the probability of an increased drain output.

## Results

### Demographic data

A total of 217 ACCF patients met inclusion criteria. Increased surgical drain output was observed in 16.6% (36 of 217) of patients. A summary of the clinical data for the patients who underwent ACCF with increased surgical drain output and the control groups is presented in Table 1. There are no significant differences in sex, BMI, preoperative systolic pressure, preoperative diastolic pressure, history of taking aspirin, and ASA classification, when the two groups were compared. The patients with increased surgical drain output was significantly older than the control group (*p* = 0.001). Of the 36 patients with increased surgical drain output, 18 patients (50.0%) were noted to have OPLL in the surgical level. However, in the control group, only 33 patients (18.2%) were noted (*p* = 0.000). Diabetes mellitus and history of smoking were also statistically associated with increased risk of increased surgical drain output: It was found in 25% (9 of 36) and 41.7% (15 of 36) of patients in the group with increased surgical drain output, as compared with 9.9% (18 of 181) and 24.3% (44 of 181) in the control group (*χ* ^2^, *p* = 0.012, *p* = 0.033, respectively). The average number of discs involved was significantly more in the increased surgical drain output group compared with that in the control group (2.42 versus 2.02, *p* = 0.001).

**Table 1:** Demographic characteristics and surgery related factors of the patients

Characteristics	Patients with increased surgical drain output		*P*
	Yes (*n* = 36)	No (*n* = 181)	
Age (years)	60.67 ± 8.18	54.41 ± 10.05	0.001
Sex, *n* (%)			0.484
Male	20(55.6)	89(49.2)	
Female	16(44.4)	92(50.8)	
BMI (kg/m^2^)	24.62 ± 4.04	25.44 ± 3.05	0.165
Preoperative systolic pressure	132.92 ± 15.14	130.99 ± 14.64	0.474
Preoperative diastolic pressure	78.25 ± 11.79	78.64 ± 11.33	0.850
OPLL in the surgical level, *n* (%)	18(50.0)	33(18.2)	0.000
Diabetes mellitus, *n* (%)	9(25.0)	18(9.9)	0.012
History of taking aspirin, *n* (%)	6(16.7)	15(8.3)	0.120
History of smoking, *n* (%)	15(41.7)	44(24.3)	0.033
ASA classification, *n* (%)			0.791
I–II	32(88.9)	158(87.3)	
III–IV	2(11.1)	23(12.7)	
Number of discs involved	2.42 ± 0.50	2.02 ± 0.65	0.001

*BMI* body mass index

### Intraoperative and postoperative data

Total drain output for this population ranged from 0 to 209 mL. The average drain output for this cohort was 29.15 ± 35.38 mL. The intraoperative blood loss was significantly more in the increased surgical drain output group compared with that in the control group (109.86 versus 87.83 mL, *p* = 0.036). The length of drainage retention in the increased surgical drain output group was longer than the control group significantly (*p* = 0.000). The increased surgical drain output group also had a significantly longer hospital stay than the control group (*p* = 0.036). However, no significant differences were found between the two groups in the hemoglobin and hematocrit values preoperatively and postoperatively (Table 2). The two groups were comparable in terms of rates of wound problem incidences (Table 3). No patient in either group underwent additional surgery for any reason.

**Table 2:** Comparing variables in patients with and without increased surgical drain output

Characteristics	Patients with increased surgical drain output		*P*
	Yes (*n* = 36)	No (*n* = 181)	
Operation time (min)	112.22 ± 16.49	105.21 ± 17.89	0.031
Intraoperative blood loss (mL)	109.86 ± 62.02	87.83 ± 56.40	0.036
Drainage (mL)	94.44 ± 37.70	16.17 ± 14.27	0.000
Length of drainage (h)	86.00 ± 25.25	50.87 ± 17.67	0.000
Hemoglobin (g/dL)			
Baseline	135.86 ± 11.60	138.27 ± 14.19	0.339
Day 1 postoperation	128.06 ± 11.65	129.33 ± 13.03	0.586
At discharge	130.92 ± 13.31	132.38 ± 15.64	0.601
Hematocrit (%)			
Baseline	39.95 ± 3.24	40.64 ± 3.83	0.314
Day 1 postoperation	37.98 ± 4.03	37.79 ± 3.83	0.791
At discharge	38.27 ± 3.81	38.95 ± 3.39	0.283
Length of stay (d)	5.17 ± 1.23	4.62 ± 1.45	0.036

**Table 3:** Comparisons of incidences of bleeding-related wound problems in patients with and without increased surgical drain output

Complications	Patients with increased surgical drain output		*P*
	Yes (*n* = 36)	No (*n* = 181)	
Dressing reinforcement, *n* (%)	0 (0)	0(0)	1.000
Oozing, *n* (%)	4(11.1)	8(4.4)	0.109
Subcutaneous hematoma, *n* (%)	0 (0)	0(0)	1.000
Ecchymosis, *n* (%)	7(19.4)	22(12.2)	0.240
Infection, *n* (%)	0 (0)	0(0)	1.000

### Predictive factors of increased surgical drain output

In the patients with increased surgical drain output group, multivariate logistic regression analysis demonstrated that age (OR, 1.075; *p* = 0.003), history of smoking (OR, 2.792; *p* = 0.021), OPLL in surgical level (OR, 2.107; *p* = 0.001), and number of discs involved (OR, 2.764; *p* = 0.003) maintained its significance in predicting likelihood of increased surgical drain output (Table 4). Nagelkerke *R* ^2^ indicated that this model explained 28.9% of the variance of likelihood of increased surgical drain output.

**Table 4:** Multivariate regression model predicting increased surgical drain output

Predictors	Odds ratio	95% confidence interval		*P* values
		Lower limit	Upper limit	
Age	1.075	1.026	1.126	0.003
History of smoking	2.792	1.168	6.678	0.021
OPLL in the surgical level	2.107	1.381	3.213	0.001
Number of discs involved	2.764	1.402	5.447	0.003

Nagelkerke *R* ^2^ = 0.289

## Discussion

A thorough understanding of the development of increased surgical drain output in cervical degenerative population treated with ACCF is a critical component to assist surgeons with the decision of whether or not to place a drain postoperatively. Although previous reports have challenged the efficacy of drains for many surgical procedures, little has assessed the necessity of drain use after ACCF [15–18]. This study compared the patients who underwent ACCF with increased surgical drain output and those who did not. The results indicate that age, OPLL in surgical level, number of discs involved, and history of smoking (highest ORs) were best predictors of increased surgical drain output for patients who underwent ACDF rather than gender, BMI, preoperative systolic pressure, diabetes mellitus history, history of taking aspirin, ASA classification, and other clinical characteristics.

It has been previously suggested that age was associated with an increased prevalence of increased drain output following spine surgery. Basques et al. found that patients with age 50 years or more were more likely to have increased drain output following ACDF and deduced that this effect may be due to delayed wound healing associated with increased age [18]. Similarly, Sokolowski et al. found that advanced age is an independent risk factor associated with postoperative hematoma volume. [19] In line with the studies of Basques et al. and Sokolowski et al., our study demonstrated that age was one of the most accurate indicators for an increased risk of increased drain output following ACCF. A possible explanation may be due to the decreased vascular elasticity and delayed wound healing with increased age, suggesting that postoperative drainage should be carefully considered in patients who underwent ACCF whose age is more than 50 years old.

Many studies have focused on the patients’ history of smoking that has been associated with many deleterious effects for surgical outcomes after musculoskeletal surgery, including decreased wound healing, increased surgical site infections, impaired fracture healing, nonunion, and increased perioperative blood loss [20–22]. In a study reported by Park et al. that involved 5280 patients undergoing single-level lumbar fusion surgery, the authors reported that smoking was a major risk factor for postoperative hematoma requiring reoperation after single-level lumbar fusion surgery [23]. In addition, smoking history has been found to increase the risk of postoperative bleeding in other neck surgery [24, 25]. This may be explained by the negative effect of smoking on the platelet membrane and its natural function. Intensive smoking cessation interventions such as individual counseling and nicotine replacement therapy administered for a period of 4 to 8 weeks before surgery seem to have the greatest effect on not only reduction of risk of complications but also increase of short and long-term cessation of smoking. Therefore, this smoking cessation program should be should be encouraged prior to ACCF.

The present study is the first report that mentions the OPLL in the surgical level as one of the independent predictors for increased surgical drain output for patients who underwent ACCF based on the analysis of the largest number of surgical subjects at a single institution. Kato et al. found that laminoplasty for OPLL is associated with a risk of major intraoperative blood loss, which can potentially give rise to devastating postoperative complications [26]. Also, Chiba et al. and Kishiya et al. compared the amount of blood loss in OPLL and cervical spondylosis myelopathy and indicated that the blood loss was greater in the OPLL group [27, 28]. We hypothesize that the bleeding tendency in patients with OPLL is caused by the abnormality of angiogenesis associated with ectopic bone formation. Therefore, we think routine use of hemostatic agents with different mechanisms of action such as absorbable gelatin sponge or tranexamic acid is of great importance in patients who underwent ACCF and who have OPLL in the surgical level to control perioperative bleeding.

The findings of this study should be viewed after considering the following limitations. Firstly, these data represent the experience at a single institution that is an academic tertiary care center with trainees in the anesthesia, orthopedic, neurosurgical, and nursing departments. Secondly, this study is a retrospective research and the results are compromised as a result of relatively limited sample and nonrandomized design. In addition, while this study describes factors predicting increased drain output, it is still not known what level of drain output can be directly linked to increased risk of clinical complications. Finally, we also acknowledge the limitations introduced by our patients’ clinical heterogeneity.

## Conclusion

The risks for increased drain output following ACCF are multifactorial. Multivariate logistic regression analysis suggests that age, history of smoking, OPLL in surgical level, and number of discs involved may be reasonable predictions for an individual likelihood of increased drain output following ACCF. The results of this study suggest that younger patients without a smoking history or OPLL who are undergoing a single-level ACCF are less likely to have increased drain output. Drains may not be necessary in this population, although further research is needed to support this conclusion. It is our hope that thru these predictive measures, the surgeon might be able to improve surgical planning, advise the patient accordingly during the consent-taking process, and apply strategies that would help in reducing the risk of increased drain output from occurring.
